# Practices of Informed Written Consent for Elective Urological Procedures at a Tertiary Care Hospital in Sudan

**DOI:** 10.7759/cureus.70235

**Published:** 2024-09-26

**Authors:** Mohammed Elsiddig, Mohammed Hassan

**Affiliations:** 1 Urology, Military Hospital, Khartoum, SDN

**Keywords:** audit, patient consent, quality improvement, surgical informed consent, urology

## Abstract

Objective

This study aimed to assess informed consent practices in elective urological surgeries at a tertiary care facility.

Materials and methods

A retrospective cross-sectional survey was carried out between March 1, 2023 and April 1, 2023, at the Department of Urology, Omdurman Military Hospital, Sudan. We included all patients who had undergone elective urological procedures under local, spinal, or general anesthesia.

The medical records were accessed to analyze the consent forms' standards. A total of 42 consent forms were included and analyzed. We use the General Medical Council's (GMC) Guidance on Professional Standards and Ethics for Doctors: Decision Making and Consent, and the Royal College of Surgeons (RCS) of England's Consent: Supported Decision-Making as the standard for our study. The GMC and the RCS of England have provided comprehensive and standardized guidelines for obtaining informed written consent, including indications, benefits, risks involved, and alternatives in addition to demographics, patient details, responsible consultant, diagnosis, and title of the surgery, intended benefits, probable risks, type of anesthesia, consenting doctor's name, designation, and signature, and the patient's signature and name.

Results

A total of 42 consent forms were included. The diagnosis and the intended surgical procedure were mentioned in all consents. The potential benefits and risks were discussed in 36 (85.7%) and 18 (42.9%) cases, respectively. The type of anesthesia was discussed in 39 (92.9%) of cases. The likely result of not having the procedure and the alternative treatment: Recorded completion rates of 10 (23.8%) and 12 (28.6%), respectively. Patient demographics were completely documented in 41 (97.6%) forms. Senior doctors were only involved in 14 (33.3%) of the consents. Details of the consenting doctor, including name, title, and signature, were present in 30 cases (71.4%), and the date of signing the consent was documented in 38 cases (90.5%). The completeness of the consent form correlated with the level of the doctor obtaining it, with consultants achieving the highest completion rates (100%), followed by registrars (66.7%) and medical officers (35.7%).

Conclusion

The current practices of informed consent were found to be substandard. Handwritten consent forms do not adhere to the recommended guidelines for informed consent in elective urological procedures. It is preferable to utilize a pre-designed consent form, allowing for personalized additions based on the patient's specifics. Our recommendation is to organize an educational session for junior doctors to emphasize proper consent procedures, and deepen their knowledge of common urological elective procedures, and associated risks. This approach promotes adherence to best clinical practices and minimizes the risk of legal challenges.

## Introduction

Informed consent is a way to educate and inform patients about their illness and the risks and benefits of the treatment they are choosing. It also includes alternative options so that they can make a knowledgeable decision about their health [1,2]. Crafted informed consent involves granting autonomous authorization and ensuring disclosure while considering the patient's ability to comprehend and provide consent. This process aims to meet both standards and legal requirements of medical practice excellence [3,4]. Consent plays a role in strengthening the bond of trust between patients and healthcare providers. It empowers patients to participate in decision-making and helps safeguard physicians and their organizations from legal issues.

The principle of consent, as outlined by the General Medical Council (GMC) in the UK, emphasizes that all patients have the right to engage in decisions regarding their treatment and care, with the necessary support to make informed choices if feasible [5]. Essential to this principle is the patient's entitlement to active listening, the provision of essential information for decision-making, and the allocation of adequate time and assistance for comprehension. Furthermore, doctors are tasked with understanding patients' concerns and preferences to effectively communicate pertinent details concerning the benefits, and risks of proposed treatments, and reasonable alternatives, which may also include the choice of taking no action [5].

Globally, medical practice has been shifted to a more patient autonomy-centered approach, yet the idea of informed consent being involved in patient autonomy is unknown to both patients and physicians in our community [6]. In Sudan, informed consent is an area of medical practice that has yet to be improved. Several factors challenge the process of obtaining valid consent, including lack of awareness, low socioeconomic status, and traditional family structure [7,8].

In our hospital, the consent form used is a generic form with spaces for the surgeon to take consent to write down the details of the procedure. The procedure is discussed with the patient, and the patient’s signature is obtained on the form. In this context, several factors can lead to incomplete filling out of the consent form. The information written in the consent form may vary depending on the level of medical professionals obtaining it. Misinterpretation or unreadable information due to the legibility of handwritten forms. Inconsistencies in terminology, including diverse medical terms or abbreviations utilized in the form, can potentially lead to insufficient or confusing information during legal proceedings. This study aimed to assess informed consent practices in elective urological procedures at a tertiary teaching hospital in Sudan in accordance with GMC, and RCS guidance.

## Materials and methods

This cross-sectional, retrospective audit aimed to evaluate the consenting practices for patients who required urological procedures between March 1, 2023, and April 1, 2023, at Omdurman Military Hospital, Sudan.

Inclusion criteria

The inclusion criteria included those patients who are above 18 years of age, irrespective of their gender, who had undergone any elective urological procedure under local, spinal, or general anesthesia between March 1, 2023 and April 1, 2023. All patients referred from the emergency department and required emergency procedures were excluded. Two authors evaluated the forms for legibility issues. Forms with unreadable words were excluded.

Data collection

The medical records were accessed to analyze the standards of the consent forms. A total of 42 consent forms were included and analyzed. We use the GMC's Guidance on Professional Standards and Ethics for Doctors: Decision Making and Consent (2020), and the Royal College of Surgeons (RCS) of England's Consent: Supported Decision-Making (2016) as the standard for our study. GMC and RCS have provided comprehensive and standardized guidelines for obtaining informed written consent, including indications, benefits, risks involved, and alternatives in addition to demographics, patient details, responsible consultant, diagnosis and title of the surgery, intended benefits, probable risks, type of anesthesia, consenting doctor's name, designation, and signature, and the patient's signature and name.

Data analysis

A Google Form (Google Inc., Mountainview, CA) was designed, containing a predefined set of the essential components of the consent form. The specific components assessed in the consent forms included: patient demographics (name, date of birth (DOB), gender), diagnosis, name of the procedure, potential benefit, potential risk, the likely result of not having the procedure, the alternative treatment, anesthesia, responsible consultant name, consenting doctor details (name, title, signature), date of signing the consent, signed by the patient or relative. Each component required a “Yes” or “No” response to facilitate straightforward analysis. Statistical Package for Social Sciences (SPSS) version 22 (IBM Corp., Armonk, NY) was used to tabulate and analyze the data. Categorical data were assessed using measures of central tendency, frequencies, and percentages. Significance was set at P < 0.05.

Ethical considerations

As the study was carried out within the urology department and was a retrospective analysis of existing documents, ethical clearance was not required.

## Results

The nature of the disease and the intended surgical procedure were completed in all consents. The potential benefits and risks were discussed in 36 (85.7%) and 18 (42.9%) cases, respectively. The type of anesthesia was discussed in 39 (92.9%) of cases. The likely result of not having the procedure and the alternative treatment: recorded completion rates of 10 (23.8%) and 12 (28.6%), respectively. Patient demographics were completely documented in 41 (97.6%). Details of the consenting doctor, including name, title, and signature, were present in 30 cases (71.4%), and the date of signing the consent was documented in 38 cases (90.5%). The completeness of the components of the consent form is shown in Table 1.

**Table 1:** Summary of the completeness of the components of the consent form

Component assessed	Number (%)
Diagnosis	42 (100)
Procedure	42 (100)
Potential benefit	36 (85.7)
Potential risk	18 (42.9)
The likely result of not having the procedure	10 (23.8)
The alternative treatment	12 (28.6)
Anesthesia	39 (92.9)
Responsible consultant name	36 (85.7)
Patient demographics (name, DOB, Gender)	41 (97.6)
Consenting doctor details (name, title, signature)	30 (71.4)
Date of signing the consent	38 (90.5)
Signed by the patient	18 (42.9)

Senior doctors were only involved in 14 (33.3%) of the consents. Twenty-eight (66.7%) consents were obtained by junior doctors, and 10 (35.7%) completed the essential components of the consent forms. The correlation between the Level of doctor obtaining consent and the completeness of the consent is shown in Table 2.

**Table 2:** Level of the doctor obtaining the consent and completeness of the consent

Level of doctor taking the consent	Number (%) of consent	Number (%) completeness of the consent form
Consultant	2 (4.8)	2 (100)
Registrar	12 (28.5)	8 (66.7)
Medical officer	28 (66.7)	10 (35.7)

## Discussion

According to the RCS of England, the act of seeking consent for surgical intervention extends beyond a mere signature on a form. It involves furnishing the necessary information that empowers the patient to decide on undergoing a particular treatment. Consent should be viewed as informed decision-making or an informed request, necessitating the investment of time, patience, and clear explanations to ensure understanding [9]. The process should provide the patient with comprehensive information about the intended surgical procedures, including indications, benefits, risks involved, and alternatives [1,2].

Our findings suggest that the quality of consent can vary depending on the experience and training of the doctor obtaining it. In this study, 28 (66.7%) consents were obtained by junior doctors, and 10 (35.7%) completed the essential components of the consent forms. On the other hand, 12 (28.5%) of the consent forms were obtained by the registrar, eight (66.7%) of them satisfied all the essential components. These findings indicate a significant correlation between the level of the doctors who obtained the consent and the completeness of the components of the consent. These findings are consistent with a previous study that showed that junior doctors who lack sufficient training faced challenges in capacity assessment, disclosure of procedure details, and consent documentation, potentially compromising the validity of acquired consent [10,11]. The limited experience of junior doctors in discussing complex medical information with patients can affect the completeness of the consent process. Also, they might still be developing these skills, which can impact how well they convey information and address patient concerns. It would be beneficial to provide them with structured communication skills training focusing on obtaining informed consent and implementing mentorship programs where junior doctors receive guidance and feedback from experienced clinicians to improve their patient interactions [11].

Consistent with a previous study, this study showed that 30 (71.4%) of the patients had no information about alternative treatments [12]. The potential benefits and risks were discussed in 36 (85.7%) and 18 (42.9%) cases, respectively. These findings are consistent with previous studies, which revealed that the risks associated with the procedures are generally vaguely described, maintaining the positive perspective and incorrect documentation on consent forms [13]. Such practice leads to invalidation of consent and puts the medical team at risk of medico-legal consequences [14,15].

The majority of the documents (57.1%) were signed by a relative of the patient, while only 42.9% were signed by the patient. This finding is valuable as it underscores the need for further studies to explore the factors that might affect the process of acquisition of valid consent, such as poverty, illiteracy, lack of awareness, low socioeconomic status, and traditional family structure [4,8].

Recommendations

We advise organizing teaching sessions and periodic training of junior healthcare professionals focused on patient communication and proper consent procedures to promote adherence to guidelines. We recommend standardization of pre-printed consent forms, which can improve documentation and compliance with guidelines. In our community, informed consent can be influenced by sociocultural variables, suggesting the need for further audits and studies to address these barriers.

Limitations

Our study's primary limitation stems from the relatively smaller sample size that might potentially impact the statistical strength and reliability of our findings. Another limitation is that the study was conducted in a single center, potentially constraining the applicability of the results to other centers.

## Conclusions

The current practices of informed consent were found to be substandard. Handwritten consent forms do not adhere to the recommended guidelines for informed consent in elective urological procedures. It is preferable to utilize a pre-designed consent form, allowing for personalized additions based on the patient's specifics. Our recommendation is to organize an educational session for junior doctors to emphasize proper consent procedures, and deepen their knowledge of common urological elective procedures, and associated risks. This approach promotes adherence to best clinical practices and minimizes the risk of legal challenges.
